# Clinical Trial Skepticism and Political Ideology Among US Adults and Cancer Survivors

**DOI:** 10.1001/jamanetworkopen.2026.9088

**Published:** 2026-04-16

**Authors:** Onyema G. Chido-Amajuoyi, Henry K. Onyeaka, Chinenye M. Okafor, Emma P. Keane, Allison O. Taylor, Crystal Okpagu, Hermioni L. Amonoo

**Affiliations:** 1 Division of Hematology and Oncology, Department of Medicine, University of Washington, Seattle; 2 Department of Psychiatry, Geisinger Health, Pennsylvania; 3 Department of Internal Medicine, Medical University of South Carolina, Florence; 4 Department of Psychiatry, Brigham and Women’s Hospital, Boston, Massachusetts; 5 Division of Hematologic Malignancies and Cellular Therapy, Department of Medicine, Duke University School of Medicine, Durham, North Carolina; 6 School of Medicine, American University of Antigua, St John’s, Antigua and Barbuda; 7 Department of Supportive Oncology, Dana-Farber Cancer Institute, Boston, Massachusetts

## Abstract

This cross-sectional study examines the association of political ideology and clinical trial skepticism in a nationally representative US sample and in a subpopulation of cancer survivors.

## Introduction

Clinical trials are essential for translating biomedical advances into improved prevention, diagnosis, and treatment. Yet, trial enrollment that ensures samples are truly representative of diverse populations remains a persistent challenge. Public willingness to enroll in clinical trials depends on trust—trust in clinicians, research institutions, and the scientific process—and recent evidence suggests political ideology shapes that trust.^1,2,3^ Therefore, we examined the association between political ideology and clinical trial skepticism in a nationally representative US sample and in a subpopulation of cancer survivors.

## Methods

This cross-sectional study followed the Strengthening the Reporting of Observational Studies in Epidemiology (STROBE) reporting guideline. We examined data from the National Cancer Institute’s Health Information National Trends Survey (HINTS) 7, conducted from March 25 to September 16, 2024. Participants in HINTS 7 reviewed and signed an electronic consent form. HINTS 7 was approved by the Westat institutional review board (IRB). HINTS data are publicly available and deidentified. Therefore, our study was exempt from IRB review per the Common Rule. The outcome of interest was clinical trial skepticism. Trial skepticism was assessed using the question, “People should be suspicious of clinical trials,” with 5 Likert-style response options ranging from strongly agree to strongly disagree. Political ideology (liberal, moderate, conservative) was the primary exposure. The HINTS 7 response rate was 27.3%, and 87.6% of respondents answered the clinical trial skepticism questions.

Multinomial logistic regression models assessed associations between mistrust or suspicion of clinical trials and political ideology in the general population and among cancer survivors. Analyses adjusted for age, sex, self-reported race or ethnicity, educational level, household income, and rural-urban residence. In sensitivity analyses, we also assessed associations using survey-weighted linear regressions. Statistical significance was defined as 2-sided *P* < .05, and analyses were performed using Stata 18.0 (StataCorp) from July to October 2025.

## Results

The overall sample of 6377 respondents included 1009 cancer survivors (9.6%), 3768 women (51.8%); 3422 non-Hispanic White individuals (60.8%); 906 non-Hispanic Black or African American individuals (10.8%); 1249 Hispanic individuals (17.4%), with 1735 participants (27.2%) agreeing that people should be suspicious of clinical trials (Table 1). Regarding political ideology, 2269 participants (37.6%) identified as moderate, 2040 (30.5%) as liberal, and 2068 (31.9%) as conservative. Cancer survivors were not significantly more likely than nonsurvivors to express suspicion toward clinical trials (neither agree nor disagree, 435 [43.5%] vs 2184 [42.2%]; strongly or somewhat agree, 204 [22.8%] vs 1529 [27.7%]; strongly or somewhat disagree, 370 [33.6%] vs 1645 [30.2%]; *P* = .13).

**Table 1.** Sample Characteristics by Political Ideology, Health Information National Trends Survey 7

Demographic variables	Total	Participant by political ideology, No. (%) [95% CI]^a^		
		Moderates	Liberals	Conservatives
Total	6377 (100)	2269 (37.6)	2040 (30.5)	2068 (31.9)
Sex				
Female	3768 (51.8)	1368 (38.8) [36.3-41.3]	1262 (32.4) [29.8-35.2]	1138 (28.8) [26.5-31.2]
Male	2547 (48.2)	878 (36.0) [33.0-39.2]	763 (29.2) [26.3-32.3]	906 (34.8) [32.0-37.7]
Age, y				
18-34	1053 (25.9)	395 (39.3) [34.4-44.4]	410 (37.1) [32.2-42.4]	248 (23.6) [20.2-27.3]
35-49	1312 (26.7)	500 (38.0) [33.2-43.2]	450 (31.8) [27.9-35.8]	362 (30.2) [26.3-34.5]
50-64	1629 (25.9)	610 (38.8) [34.8-42.9]	458 (26.2) [22.8-29.8]	561 (35.0) [31.6-38.6]
≥65	2332 (21.5)	745 (32.8) [29.7-36.0]	709 (26.8) [24.3-29.5]	878 (40.4) [37.2-43.7]
Education				
High school or less	1408 (26.6)	602 (40.6) [36.0-45.5]	332 (26.6) [22.3-31.4]	474 (32.8) [28.9-36.9]
Some college	1842 (38.6)	737 (42.3) [38.4-46.2]	452 (25.6) [22.1-29.4]	653 (32.1) [28.2-36.4]
College graduate or more	3101 (34.8)	918 (29.8) [26.9-32.9]	1251 (39.2) [35.9-42.6]	932 (31.0) [28.5-33.6]
Household income				
<$20 000	1026 (14.5)	432 (44.6) [38.7-50.5]	260 (25.2) [20.1-31.0]	334 (30.3) [24.0-37.3]
$20 000-$34 999	747 (11.2)	313 (43.0) [36.5-49.6]	207 (30.5) [24.1-37.7]	227 (26.5) [20.3-33.8]
$35 000-$49 999	755 (11.6)	292 (40.3) [34.5-46.5]	205 (24.2) [19.9-28.9]	258 (35.5) [30.1-41.3]
$50 000-$74 999	1001 (15.7)	355 (43.5) [38.2-49.0]	331 (27.7) [22.9-33.1]	315 (28.7) [24.5-33.4]
≥$75 000	2598 (47.0)	789 (31.6) [28.2-35.2]	984 (35.2) [32.1-38.4]	825 (33.3) [30.9-35.7]
Race^b^				
Hispanic	1249 (17.4)	493 (40.6) [35.9-45.5]	361 (32.1) [27.3-37.4]	395 (27.2) [23.5-31.4]
Non-Hispanic Black or African American	906 (10.8)	422 (50.7) [44.4-56.9]	303 (27.5) [23.5-31.9]	181 (21.9) [16.9-27.8]
Non-Hispanic White	3422 (60.8)	1026 (33.3) [30.0-36.6]	1125 (30.5) [27.4-33.7]	1271 (36.3) [33.5-39.1]
Other^c^	578 (11.0)	237 (44.2) [36.7-52.0]	188 (30.6) [24.0-38.1]	153 (25.2) [18.9-32.7]
Residence				
Urban	5495 (87.2)	1978 (37.9) [35.3-40.6]	1836 (32.0) [29.5-34.5]	1681 (30.1) [28.2-32.2]
Rural	882 (12.8)	291 (35.3) [30.6-40.3]	204 (20.8) [17.4-24.8]	387 (43.9) [38.9-49.0]
Clinical trial skepticism^d^				
Neither agree or neither disagree	2623 (42.3)	1048 (39.9) [36.3-43.6]	697 (26.7) [23.4-30.4]	878 (33.4) [30.1-36.8]
Strongly or somewhat agree	1735 (27.2)	635 (42.4) [38.0-46.9]	447 (21.8) [18.2-25.9]	653 (35.8) [32.1-39.7]
Strongly or somewhat disagree	2019 (30.5)	586 (30.0) [27.0-33.3]	896 (43.6) [39.7-47.6]	537 (26.4) [23.5-29.5]
Cancer status				
No	5358 (90.4)	1948 (38.0) [35.4-40.7]	1725 (30.9) [28.6-33.3]	1685 (31.1) [29.2-33.1]
Yes	1009 (9.6)	315 (33.2) [28.2-38.6]	312 (27.3) [23.3-31.7]	382 (39.5) [34.1-45.1]

^a^ Political ideology was derived with the survey question, “Thinking about politics these days, how would you describe your own political viewpoint?” Responses were categorized into 3 groups. The liberal category included very liberal, liberal, and somewhat liberal. The conservative category included very conservative, conservative, and somewhat conservative. The final category was moderate.

^b^ Race was assessed as part of the sociodemographic characteristics of study participants and was self-reported.

^c^ No further breakdown of the other category was available from the National Cancer Institute Health Information National Trends Survey.

^d^ Participants were asked to assess their mistrust or suspicion of clinical trials with the following prompt: “People should be suspicious of clinical trials.” Response options included strongly agree, somewhat agree, neither agree nor disagree, somewhat disagree, and strongly disagree. Strongly agree and somewhat agree were collapsed into a single response category, and strongly disagree and somewhat disagree were also collapsed into a single response category. Response neither agree nor disagree was used as the reference category.

Compared with respondents who neither agreed nor disagreed, conservatives were more likely to agree with the statement “People should be suspicious of clinical trials” than liberals (aOR, 1.30; 95% CI, 1.10-1.54) and moderates (aOR, 1.43; 95% CI, 1.22-1.67) (Table 2). Liberals were more likely than moderates to disagree with the statement “People should be suspicious of clinical trials” (aOR, 1.98; 95% CI, 1.70-2.31).

**Table 2.** Association Between Clinical Trial Skepticism and Political Ideology in the US Overall Population and Cancer Survivors

Level of agreement with the statement “People should be suspicious of clinical trials”^a^	Political ideology, aOR (95% CI)^b^^,^^c^		
	Conservative vs liberal	Conservative vs moderate	Liberal vs moderate
**Overall US population (n = 5854)**			
Neither agree or nor disagree	1 [Reference]	1 [Reference]	1 [Reference]
Strongly agree or somewhat agree	1.30 (1.10-1.54)	1.43 (1.22-1.67)	1.10 (0.93-1.30)
Strongly disagree or somewhat disagree	0.48 (0.41-0.56)	0.95 (0.81-1.12)	1.98 (1.70-2.31)
**Cancer survivors (n = 1009)**			
Neither agree or nor disagree	1 [Reference]	1 [Reference]	1 [Reference]
Strongly agree or somewhat agree	1.34 (0.82-2.19)	1.72 (1.11-2.69)	1.28 (0.77-2.13)
Strongly disagree or somewhat disagree	0.44 (0.30-0.66)	1.06 (0.72-1.58)	2.39 (1.60-3.57)

Abbreviation: aOR, adjusted odds ratio.

^a^ Mistrust or suspicion of clinical trials was assessed using the statement: “People should be suspicious of clinical trials.” Response options included “strongly agree,” “somewhat agree,” “neither agree nor disagree,” “somewhat disagree,” and “strongly disagree.” “Strongly agree or somewhat agree” were collapsed into agree; “strongly disagree or somewhat disagree” were collapsed into disagree; “neither agree nor disagree” was the reference category.

^b^ Political ideology was derived from: “Thinking about politics these days, how would you describe your own political viewpoint?” Very liberal, liberal, or somewhat liberal were categorized as “liberal”; very conservative, conservative, or somewhat conservative were categorized as “conservative”; the remaining category was “moderate.”

^c^ aORs were adjusted for age, sex, race and ethnicity, educational level, household income, and rural-urban residence.

 Compared with respondents who neither agreed nor disagreed, conservative cancer survivors were more likely to agree with the statement “People should be suspicious of clinical trials” compared with moderate cancer survivors (aOR, 1.72; 95% CI, 1.11-2.69), and less likely to disagree compared with liberal cancer survivors (aOR, 0.44; 95% CI, 0.30-0.66) (Table 2). Liberal cancer survivors were more likely than moderate survivors to disagree with the statement “People should be suspicious of clinical trials” (aOR, 2.39; 95% CI, 1.60-3.57).

## Discussion

In this cross-sectional study, political ideology was associated with clinical trial skepticism, with conservative identification corresponding to higher odds of trial skepticism. Among cancer survivors, results were similarly suggestive of greater trial skepticism among respondents who identified as conservative, although subgroup estimates were less precise. This pattern is consistent with broader polarization in trust toward scientific institutions^3,4^ and government health agencies,^5,6^ as well as variable participation in clinical trials.^3^ Limitations include cross-sectional design with no causal inference, a single-item measure of suspicion, and potential nonresponse bias (HINTS response rate, 27.3%).

Implications are both pragmatic and urgent. If political ideology influences receptivity to clinical trials, recruitment strategies that overlook ideological diversity risk biased enrollment and underrepresentation. Prior research demonstrates disparities in clinical trial participation by political orientation.^2^ Recognizing political ideology as a factor in medical mistrust may help reduce participation barriers and improve representativeness.

Tailored engagement strategies should acknowledge and address ideological heterogeneity. Interventions could include ideologically congruent messaging, clinician-mediated invitations emphasizing local oversight and patient confidentiality, efforts to diversify clinical trialists, and community partnerships that build credibility across political groups.

## Notes

Supplement.
Data Sharing Statement
