# Supplementary material for: Clinical Trial Skepticism and Political Ideology Among US Adults and Cancer Survivors
Source: JAMA Netw Open. 2026 Apr 16;9(4):e269088. doi: 10.1001/jamanetworkopen.2026.9088 (PMC13087811; doi:10.1001/jamanetworkopen.2026.9088)
Supplement: Supplement. — Data Sharing Statement [file jamanetwopen-e269088-s001.pdf]

# Data Sharing Statement

Chido-Amajuoyi. Clinical Trial Skepticism and Political Ideology Among US Adults and Cancer Survivors. *JAMA Netw Open*. Published April 16, 2026.
doi:10.1001/jamanetworkopen.2026.9088

**Data**
**Data available:** Yes
**Data types:** Deidentified participant data
**How to access data:** Data Sharing Statement: Original data from the National Cancer Institute's Health Information National Trends Survey (HINTS) 7 can be found at https://hints.cancer.gov/.
**When available:** With publication

**Supporting Documents**
**Document types:** None

**Additional Information**
**Who can access the data:** Publicly available data
**Types of analyses:** For any purpose
**Mechanisms of data availability:** Publicly available data
